# STARgrid: an open-source pipeline from Visium HD sequencing reads to count matrices

**DOI:** 10.64898/2026.10.09.757882

**Published:** 2026-10-12

**Authors:** Ling-Hong Hung, Ka Yee Yeung

## Abstract

In Visium HD, transcript positions are computed from sequenced barcodes, which are matched to a list of coordinate barcodes that map to a grid of capture locations. Quantifying reads and assigning their positions have relied on Space Ranger, whose end-user licence prohibits modification and whose published source cannot be redistributed. We present STARgrid, the first open-source pipeline that takes Visium HD FASTQ files from Flex or RNA-seq based quantitation to count matrices at 2, 8 and 16 µm; it is part of STAR Suite. On three human slides its output agrees closely with Space Ranger’s (correlations of gene totals are at least 0.9989), and STARgrid takes 20–35% less wall time under the tested configurations. STARgrid assigns each read barcode to the coordinate barcode with the most similar sequence, as assessed by edit distance. In an examination of Space Ranger’s per-read output on one further slide, Space Ranger does the same for most reads, with two differences. First, where two coordinate barcodes overlap, the shorter being the longer without its first base, Space Ranger records the shorter barcode, although the longer one matches with fewer edits; in all, Space Ranger’s location is not among the closest for 0.5980% of the reads that our decoder can place. Second, Space Ranger settles each read on its own, recording one location or none, and leaves 0.2916% of these reads without a location; STARgrid keeps every matching location and chooses among them for each molecule, using all its reads. Our molecule totals are 1.9–7.6% above Space Ranger’s. The larger source of this difference is probably that we resolve location for each molecule and not for each read; STAR Suite’s own gene assignment and molecule counting may also contribute. The counts that STARgrid adds behave like signal. On H&E images of the same sections they fall in cells at nearly the rate of the counts that both pipelines report. Against an adjacent Xenium section of one slide, our counts are closer to Xenium than Space Ranger’s for 15 of the 20 genes of a cancer gene panel that Xenium measures most abundantly ( *P* = 0.0414). The added counts therefore appear to improve the measurement of cancer-associated expression. These results are preliminary, but the method is now open, and every comparison reported here can be repeated, extended and improved upon.

